# High prevalence and diversity of Bartonella in small mammals from the biodiverse Western Ghats

**DOI:** 10.1371/journal.pntd.0009178

**Published:** 2021-03-11

**Authors:** B. R. Ansil, Ian H. Mendenhall, Uma Ramakrishnan

**Affiliations:** 1 National Centre for Biological Sciences, Tata Institute of Fundamental Research, Bangalore, Karnataka, India; 2 Manipal Academy of Higher Education, Manipal, Karnataka, India; 3 Duke-National University of Singapore Medical School, Programme in Emerging Infectious Diseases, Singapore; University of Pretoria, SOUTH AFRICA

## Abstract

*Bartonella* species are recognized globally as emerging zoonotic pathogens. Small mammals such as rodents and shrews are implicated as major natural reservoirs for these microbial agents. Nevertheless, in several tropical countries, like India, the diversity of *Bartonella* in small mammals remain unexplored and limited information exists on the natural transmission cycles (reservoirs and vectors) of these bacteria. Using a multi-locus sequencing approach, we investigated the prevalence, haplotype diversity, and phylogenetic affinities of *Bartonella* in small mammals and their associated mites in a mixed-use landscape in the biodiverse Western Ghats in southern India. We sampled 141 individual small mammals belonging to eight species. *Bartonella* was detected in five of the eight species, including three previously unknown hosts. We observed high interspecies variability of *Bartonella* prevalence in the host community. However, the overall prevalence (52.5%) and haplotype diversity (0.9) was high for the individuals tested. Of the seven lineages of *Bartonella* identified in our samples, five lineages were phylogenetically related to putative zoonotic species–*B*. *tribocorum*, *B*. *queenslandensis*, and *B*. *elizabethae*. Haplotypes identified from mites were identical to those identified from their host species. This indicates that these *Bartonella* species may be zoonotic, but further work is necessary to confirm whether these are pathogenic and pose a threat to humans. Taken together, these results emphasize the presence of hitherto unexplored diversity of *Bartonella* in wild and synanthropic small mammals in mixed-use landscapes. The study also highlights the necessity to assess the risk of spillover to humans and other incidental hosts.

## Author summary

Zoonotic bacterial infections cause both economic and health burdens to humans, especially in developing countries. *Bartonella* is a diverse group with several species that are infectious to humans. There is limited knowledge of *Bartonella* diversity and subsequent risk for spillover in several tropical countries, including India. This study, set in a biodiversity hotspot with high human density, reveals a high prevalence and diversity of *Bartonella* in the most common, and synanthropic small mammals. Several lineages identified are phylogenetically related to zoonotic species known to cause infections in humans. We suggest that there is unexplored diversity of *Bartonella* in small mammals that reside in human-modified landscapes in India, demonstrating an urgent need for further investigation. By identifying the natural reservoirs of pathogens, their ecology, and their relationship and proximity to humans, we will be able to proactively survey for these under-detected bacterial infections and minimize the burden.

## Introduction

The genus *Bartonella* comprises many cosmopolitan species capable of infecting a wide range of mammals, including humans [1]. Of the approximately 46 defined species, at least 18 are zoonotic [2,3]. Small mammals such as rats, squirrels, and shrews act as reservoirs for several of these bacterial species [1], while hematophagous arthropods, such as ticks, mites, and fleas transmit these pathogens between reservoirs and incidental hosts [4]. Infections in humans include cat scratch fever (*Bartonella henselae*), trench fever (*B*. *quintana*), and carrion’s disease (*B*. *bacilliformis*) [5–7]. Symptoms of these infections present as swollen lymph nodes (lymphadenopathy) with prolonged fever, presence of bacteria in red blood cells (intraerythrocytic bacteremia), inflammation of the optic nerve and retina (neuroretinitis), and often infections of the heart’s inner lining (endocarditis) [2].

Despite the evidence of disease burden in humans, bartonellosis remains under-detected in tropical countries such as India. Few studies investigate *Bartonella*-associated infections in humans in India [8–10]. For instance, in the Government General Hospital, Chennai, in South India (2005–2006), patients admitted with endocarditis were diagnosed with *B*. *quintana* [10]. Similarly, in tertiary care hospitals in North India, *B*. *henselae* was detected in patients admitted with fever, lymphadenopathy, and optic neuritis [8,9]. All these studies reported a high prevalence (8–23%) of *Bartonella* indicating that bartonellosis is an important existing infection. Other than these studies, there is no information available on the prevalence or the burden of this infection in India. Even more poorly documented are the diversity, natural maintenance, incidental hosts, vectors, and mode of transmission of these pathogens. We seek to bridge this gap by investigating the presence of *Bartonella* species in small mammal hosts in India.

As a group, small mammals primarily consist of non-volant mammals such as rodents and shrews that usually weigh less than 1 kg [11]. They host several medically important zoonotic pathogens, such as viruses (Hantaviruses, Lassa virus) bacteria (Lyme disease, leptospirosis), protozoans (toxoplasmosis), and helminths (rat lungworm) [12,13]. Their high birth and growth rates and synanthropic nature facilitate high rates of contact with humans and provide opportunities for spillover [14]. Their cosmopolitan distribution and increased connectivity, especially because of human facilitated dispersal, enables the influx and efflux of host and pathogen gene flow, posing a serious challenge for public health [15].

The potential risk of zoonoses from small mammals is very significant in a country like India, which is home to 101 species of rodents and 30 species of shrews [16]. Along with the high richness of small mammals, India is also experiencing high rates of deforestation and associated land-use change [17]. Natural habitats in the country are often interspersed with agricultural fields, plantations, and habitations with high human density [18,19]. This spatial structuring leads to increased human-livestock-wildlife interactions [20], which alongside high density of generalist and synanthropic small mammals poses a higher risk of zoonotic infections [12,21]. Therefore, identifying pathogens in their natural reservoirs and vectors is imperative for understanding their transmission dynamics, particularly in rapidly changing landscapes.

In this study, we investigated the presence of *Bartonella* in small mammal species and associated mites from a mixed-use landscape in the biodiverse Western Ghats in the southern Indian state of Karnataka. We examined the prevalence and haplotype diversity of *Bartonella* species in the small mammal host community. Studies from other tropical countries find high *Bartonella* prevalence in small mammal communities [22]. They also find that among different groups of small mammals in these communities, synanthropic species tend to have high prevalence and diversity [22,23]. In accordance with these patterns from other tropical small mammal communities, we expected to find (a) high overall prevalence and (b) high prevalence and diversity in synanthropes. We used PCR-based screening, multi-locus sequencing, and phylogenetic methods to investigate these predictions and understand the phylogenetic affinities of *Bartonella* in small mammals and their ectoparasites. Using this information, we aim to identify natural reservoir hosts and vectors of these emerging pathogens to ultimately better understand the risk of zoonotic spillover.

## Methods

### Ethical statement

This study was approved by the National Centre for Biological Sciences (NCBS) Institutional Animal Ethics Committee (UR-5/2014 and NCBS-IAEC-2016/10-[M]). Sample collection and processing in this study adhered to strict biosafety guidelines and approved by the Institutional Biosafety Committee (TFR: NCBS:23_IBSC/2017). Field sampling in Kadumane Tea Estate was conducted under the provision of a Memorandum of Understanding (MoU) signed between Kadumane Estate Company and NCBS.

### Small mammal trapping and sample collection

**Fig 1:**
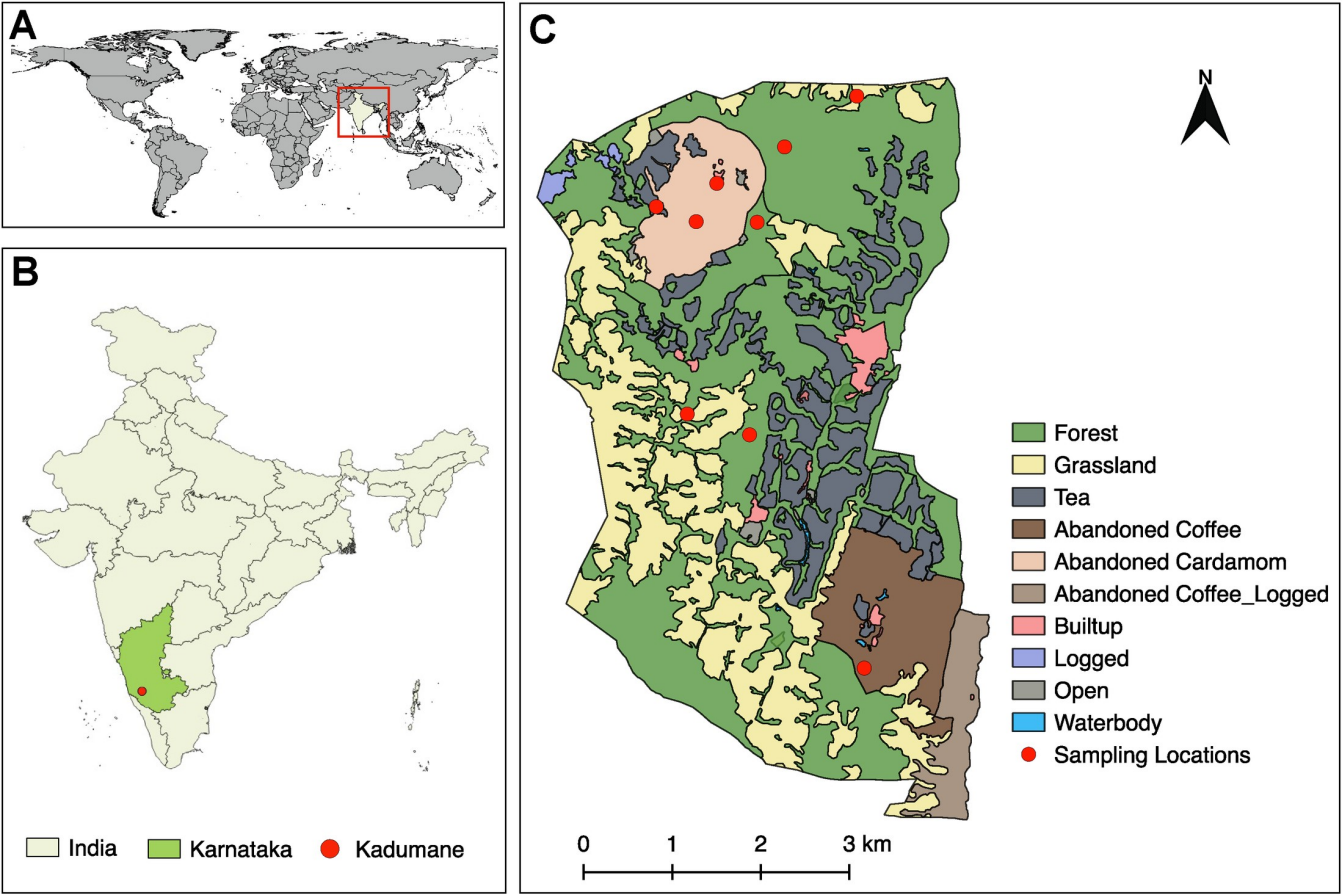
Map of the study area and sampling sites. (A) World map with India marked inside the red box. (B) Map of India with the Karnataka state and the Kadumane landscape marked. (C) The enlarged land-use land cover map of Kadumane. Red circles indicate sampling locations. The shapefile for the world was downloaded from DIVA-GIS (http://www.diva-gis.org/gdata) and the shapefile for India from Community Created Maps of India (http://projects.datameet.org/maps/). The land-use map of Kadumane was created by Nature Conservation Foundation. All layers are in WGS84 geographic coordinate system and were processed and plotted using QGIS3.10 (https://qgis.org/en/site/), an open-access software.

This study was part of a larger project investigating small mammals and associated bacterial communities in mixed-use landscapes in the Western Ghats. Small mammal trapping was conducted during the dry months (January–May) of 2016, 2017, and 2018 in different land-use types present in the Kadumane Tea Estate, located in the state of Karnataka in South India. Land-use types in the study area included forest fragments, grasslands, abandoned plantations, tea plantations, and human habitations (Fig 1). Nine sites were selected across these land-use types for small mammal trapping. The number of sites in each land-use type was decided based on the available area. Each site was sampled for small mammals using a grid framework, comprising 100 medium-sized Sherman traps (H.B. Sherman Traps, Inc., Tallahassee FL, USA) arranged in 10 lines. Each grid was sampled for four consecutive days, for a total of 400 trap nights per site. In forests and abandoned plantations, 20 additional traps were placed (a total of 480 trap nights per site) on trees at different heights to capture arboreal small mammals. Traps were baited with peanut butter and checked twice a day (morning and evening) for captures. Species were identified with the help of taxonomic keys and field guides [24,25].

Individuals captured were humanely sacrificed by overexposure to isoflurane (Baxter International, Deerfield, IL, USA) and dissected for blood and spleen collection. Cardiac blood was collected on Whatman FTA cards (GE Life Sciences, Pittsburgh, PA, USA) and stored using silica gel as a desiccant. Spleen samples were collected in RNAlater (Sigma-Aldrich, St. Louis, MO, USA) and stored at 4°C for one week before being transferred to a -20°C freezer at the National Centre for Biological Sciences (NCBS) in Bangalore. From the small mammals captured, individuals from which either spleen or blood was collected were used. Hematophagous mites were collected by combing each individual over a sheet of white paper placed in a tray and stored in 100% ethanol. An average of 5 mites (ranging from 2–10) was collected from each individual and identified morphologically using a stereomicroscope. The shape of the genital ventral plate, anal plate, and mouthpart morphology were used as identification features based on illustrations provided in previous studies and taxonomic keys [26–28].

### PCR-based *Bartonella* screening

Approximately 25 mg of spleen was homogenized using Silicon-Carbide Sharp Particles in Mini-Beadbeater-96 bead beater (BioSpec Products, Bartlesville, OK, USA) and DNA was extracted using ZR-Duet DNA/RNA Mini-Prep Plus Kit—D7003 (Zymo Research Corporation, Irvine, CA, U.S.A.). For mite DNA extraction, 5–10 individuals were pooled by host species and the land-use type and processed as above. Considering the microscopic size and large number, pooling was an ideal method to yield enough DNA for *Bartonella* detection. For DNA extraction from dried blood spots, 3 mm diameter circles (n = 3–5 circles) were cut out from FTA cards and extracted using a QIAamp DNA Mini Kit (QIAGEN, Hilden, Germany). DNA extracts from the spleen, dried blood spots, and pooled mites were subjected to an initial screening PCR targeting an 825 bp region of the *rpoB* locus (beta subunit of bacterial RNA polymerase) of *Bartonella* [29]. The 25 μl PCR reaction mixture was comprised of 2 μl of template DNA, 12.5 μl of 1X HotStarTaq Master Mix (QIAGEN, Hilden, Germany), 1 μl each of 5 μM primers, and 8.5 μl of nuclease-free water. For screening dried blood spots, 4 μl of template DNA was used to increase PCR detection. Nuclease free water and DNA extraction controls were used as negative controls for PCR reactions. Previously identified positive *Bartonella* samples confirmed by sequencing were used as positive controls. PCR products were visualized on a 1.5% agarose gel stained with GelRed Nucleic Acid Gel Stain (Biotium Inc., Fremont, CA, USA). Amplified products were purified using AMPure XP magnetic beads (Beckman Coulter, IN, USA) and sequenced at the NCBS Genomics facility. Sequences generated were inspected using Geneious 11.1.4 (Biomatters, Ltd., Auckland, New Zealand) to assess quality and to trim primer binding sites and low-quality ends. These sequences were examined for similarity with *Bartonella* using the BLAST algorithm at NCBI [30]. In addition, a ~900 bp region of the *ftsZ* (cell division protein) and a 369 bp region of the *16S* rRNA (16S ribosomal RNA) loci were amplified for each *rpoB*-positive sample [31,32]. PCR products for *ftsZ* and *16S* were visualized, purified, and sequenced as described earlier. PCR primer sequences and annealing temperature for all three loci are provided in the S1 Table. GenBank accession numbers of sequences generated in this study are provided in the S2 Table.

### Prevalence estimation

From the PCR screening results, the number of positives, negatives, and the total number of individuals were summarized and counted for each species using the ‘dplyr’ package in R [33]. The presence of *Bartonella* in either the spleen or the blood sample was considered a positive individual. From these data, species-specific and overall prevalence was calculated as the proportion of positive individuals in the total number of individuals tested. We estimated 95% confidence intervals around the prevalence for the four most common species. These confidence intervals are the binomial probabilities of positives (success) calculated from the sample (n) which include positives and negatives (success and failure). We used Wilson’s method [34] included in R Package ‘HMisc’ [35] for calculating these values. Confidence interval estimation was not possible for other species as they did not meet the minimum sample size requirement (10 individuals). The result of the same was plotted using ggplot2 [36].

### Haplotype diversity and phylogenetic analysis

A consensus of forward and reverse sequences was generated for the partial *rpoB* gene to construct a haplotype network and calculate haplotype diversity. Only sequences with an initial quality score greater than 70% were used in the analysis. By setting this cutoff, we were able to remove low-quality mixed sequences from our sequence datasets. The sequence quality scores increased to 99–100% upon trimming primer binding sites, and low-quality ends. We also verified that no degenerate bases were present in the alignment created for the haplotype network analysis. Aligned *rpoB* sequences were imported to POPART [37] and an integer neighbor joining network was created. Names of the corresponding host species were used as traits and mapped to the alignment during the analysis. Haplotype diversity was calculated using the R package ‘pegas’ [38]. The corresponding host of each haplotype was identified from the haplotype network and used to construct a presence-absence matrix. A haplotype accumulation curve was created based on this presence-absence matrix and extrapolated to estimate the potential number of haplotypes that could be detected from different small mammal species present in the landscape. R package ‘vegan’ [39] and ‘iNEXT’ [40] were used for these analyses.

In order to construct the phylogeny, *rpoB* sequences of other known *Bartonella* species were downloaded from the NCBI database and a 773 bp alignment was created using MUSCLE in MEGA 7.0.26 [41]. Concatenated alignments of *rpoB*, *ftsZ*, and *16S* sequences (1,861bp) were created following the same procedure. Alignments were codon-optimized (except *16S*) and tested for the appropriate nucleotide substitution models using PartitionFinder2 [42]. As Bayesian methods only allow partitioned heterogenous substitution models, we used BEAST 1.10.0 [43] for phylogenetic analysis. *Brucella melitensis* was selected as an outgroup and each phylogeny was run for 10^8^ Markov chain Monte Carlo (MCMC) cycles. Effective sample sizes and parameter convergence of MCMC sampling were assessed using Tracer v1.6 [44]. The resulting trees were used to generate a consensus tree (after 25% burnin), which was visualized and annotated using FigTree 1.4.3 [45]. Internal nodes of the tree are marked and colored based on the Posterior Probability (PP).

## Results

### Small mammal assemblage and *Bartonella* prevalence

Blood or spleen samples were collected from 141 individuals belonging to eight species captured across five different land-use types (Table 1). *Rattus satarae*, *Rattus rattus*, *Mus booduga*, and *Mus musculus* were the most common species in the captures. Among these, *R*. *satarae* (n = 67) was the most abundant, followed by *M*. *booduga* (n = 35), and *M*. *musculus* (n = 24). *R*. *satarae* was captured in forests and abandoned plantations, while *R*. *rattus* (n = 10) was only captured in human habitation. Although *M*. *booduga* was abundant in grasslands (n = 28), a few individuals were also trapped in forests (n = 2) and human habitations (n = 5). *M*. *musculus* was trapped largely in grasslands (n = 21) and rarely in forests (n = 3). *Golunda ellioti* (n = 1) was only trapped in grassland. Species such as *Funambulus tristriatus* (n = 1) and *Platacanthomys lasiurus* (n = 1) were rare and trapped only in forests. In addition to *R*. *rattus* and *M*. *booduga*, *Suncus murinus* (n = 2), was also trapped in human habitation. No small mammals were trapped in tea plantations. From these small mammals, ~250 mites were collected and identified to the family Macronyssidae, a family that includes tropical rat mites. All the mites collected from different small mammal species were morphologically identical. This could be due to the cryptic life stage morphology of these taxonomically complex groups. Therefore, to avoid erroneous taxonomic assignments, we report them as ’mites’ henceforth.

**Table 1:** Prevalence of *Bartonella* in small mammals captured in different land-use types in the study area.

	Prevalence (%)					
Small mammal species	Forest	Grassland	Abandoned plantation	Tea plantation	Human habitation	Total
*Rattus satarae*	24/29 (82.75)	-	33/38 (86.84)	-	-	57/67 (85.07)
*Rattus rattus*	-	-	-	-	0/10 (0)	0/10 (0)
*Mus booduga*	1/2 (50)	0/28 (0)	-	-	0/5 (0)	1/35 (2.85)
*Mus musculus*	2/3 (66.66)	12/21 (57.14)	-	-	-	14/24 (58.33)
*Golunda ellioti*	-	0/1 (0)	-	-	-	0/1 (0)
*Platacanthomys lasiurus*	0/1 (0)	-	-	-	-	0/1 (0)
*Funambulus tristriatus*	1/1 (100)	-	-	-	-	1/1 (100)
*Suncus murinus*	-	-	-	-	1/2 (50)	1/2 (50)
**Total**	28/36 (77.77)	12/50 (24)	33/38 (86.84)	0/0 (0)	1/17 (5.88)	74/141 (52.48)

Prevalence is reported as the percentage of individuals that tested positive for *Bartonella* in each land-use type.

**Fig 2:**
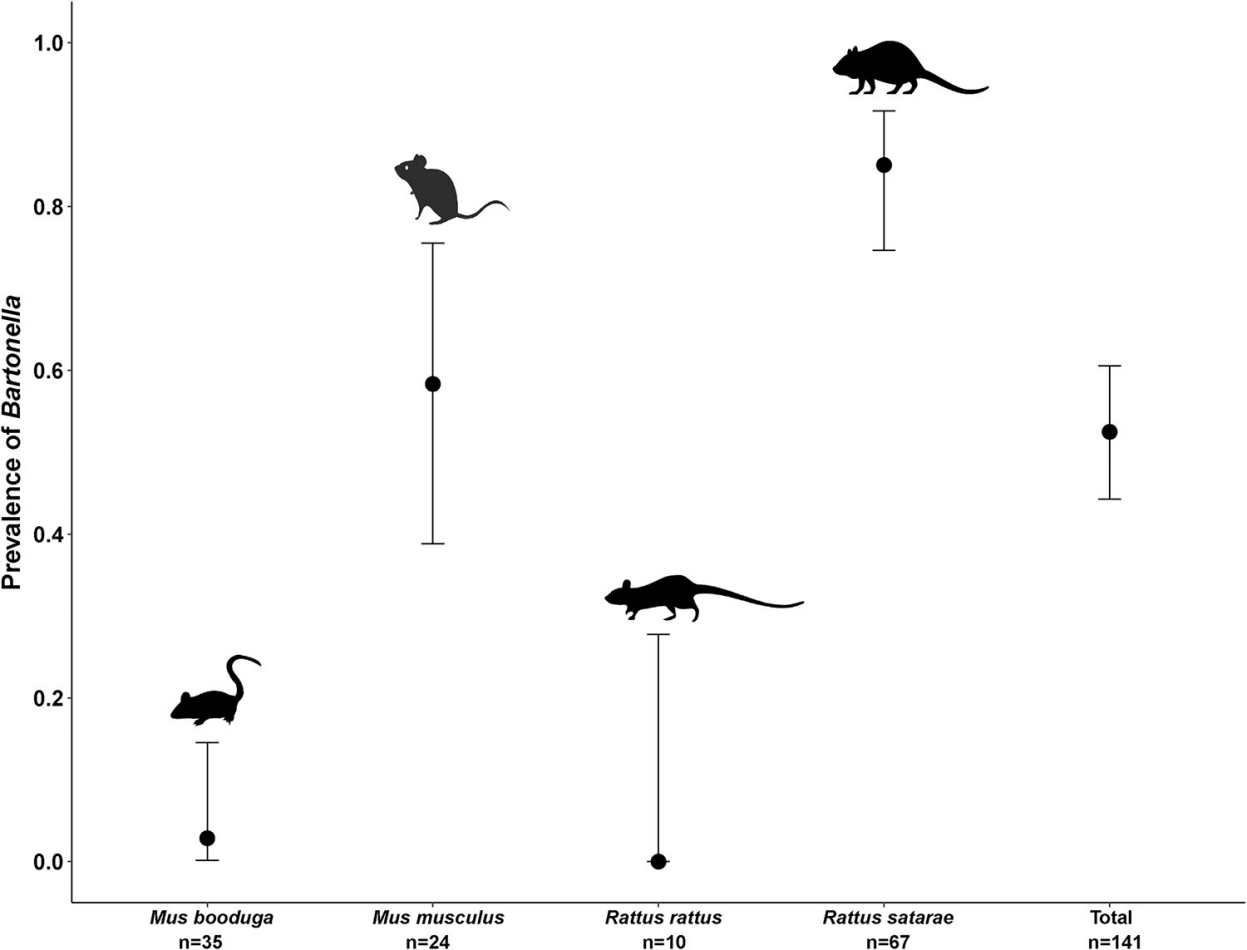
Prevalence of *Bartonella* in the four most common small mammal species. This figure summarizes our predictions about the high prevalence of *Bartonella* in the small mammal community. The dots represent calculated prevalence and the bar represents 95% confidence intervals. The sample size of the other species was too low to calculate confidence intervals. The silhouette images of animals were downloaded from PhyloPic (http://phylopic.org), an open-access database that stores reusable silhouette images of organisms.

*Bartonella* was present in 52.48% of the small mammals tested (Table 1). Among the most common four species, *R*. *satarae* had the highest prevalence of *Bartonella* at 85.07% (n = 67) (Fig 2). Synanthropic species *M*. *musculus* had a prevalence of 58.33% (n = 24), followed by *M*. *booduga* at 2.85% (n = 35), a species abundant in grasslands but often present in forests and human habitations. All the *R*. *rattus*, another well-recognized synanthrope, screened were PCR-negative for *Bartonella*. The only individual of *F*. *tristriatus* caught was positive while one of the two *S*. *murinus* individuals was positive. Screening results obtained from dried blood spots were consistent with the spleen samples (Table 2). Of the seven mite pools screened, one pool from *M*. *musculus* (n = 3) and another one from *M*. *booduga* (n = 1) was positive. Other mite pools from *R*. *satarae*, *G*. *ellioti*, and *P*. *lasiurus* were negative. A list of all individual small mammals used in this study and *Bartonella* screening results for spleen and dried blood spots are provided in S1 File.

**Table 2:** Tissue specific prevalence of *Bartonella* in different species of small mammals in the study area.

	Prevalence (%)		
Small mammal species	Spleen	Dried bloodspot	Pooled mites
*Rattus satarae*	57/67 (85.07%)	50/66 (75.75)	0/1 (0%)
*Rattus rattus*	0/10 (0%)	0/10 (0%)	-
*Mus booduga*	1/35 (2.85%)	1/32 (3.12)	1/1 (100%)
*Mus musculus*	11/24 (45.83)	12/22 (54.54)	1/3 (33.33%)
*Golunda ellioti*	0/1 (0%)	-	0/1 (0%)
*Platacanthomys lasiurus*	0/1 (0%)	-	0/1 (0%)
*Funambulus tristriatus*	1/1 (100%)	-	-
*Suncus murinus*	1/2 (50%)	0/2 (0%)	-
**Total**	71/141 (50.35)	63/132 (47.72)	2/7 (28.57%)

For each sample type, prevalence is reported as the percentage of samples that tested positive for *Bartonella*. Mites collected were pooled for screening based on host species. Hence, the numerator represents the number of positive pools and the denominator represents the total number of pools tested.

### Haplotype diversity and host association of *Bartonella*

**Fig 3:**
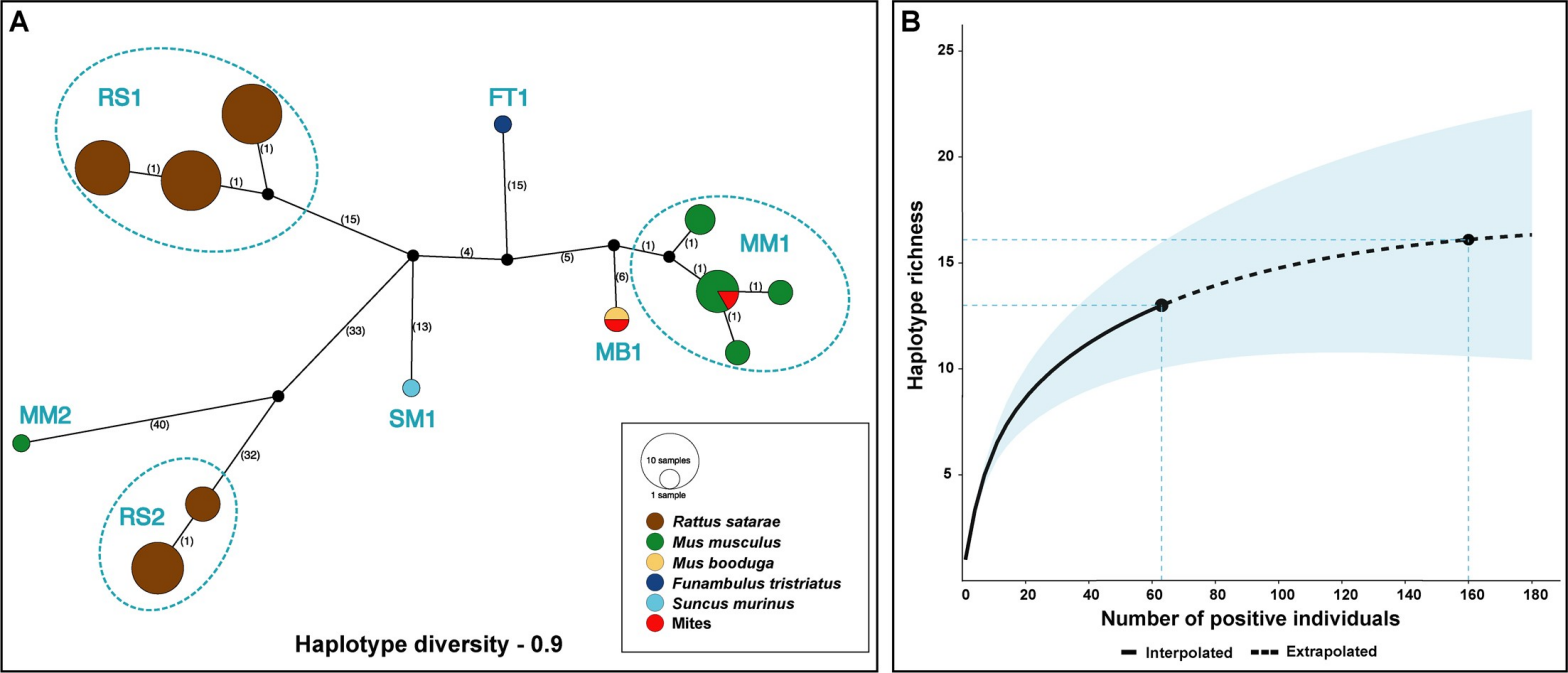
Haplotype diversity. (A) Haplotype network showing the relationship among *Bartonella* sequences from small mammal host species in Kadumane, South India. An integer neighbor joining network was constructed using 657 bp of *rpoB* using PopArt. The numbers between nodes indicate the number of nucleotide differences between haplotypes. (B) The haplotype accumulation curve (solid line) shows the cumulative haplotype richness (*rpoB*) sampled across 63 positive small mammals. The extrapolated richness (dotted lines) saturates around the estimated requirement to sample 160 positive individuals to detect all possible haplotypes in this community.

Of the 74 PCR-positives, 65 *rpoB* sequences (63 sequences from small mammals and two sequences from pooled mite samples) met quality thresholds and were used to construct the haplotype network and calculate haplotype diversity. Analysis of 657 bp of these *rpoB* sequences revealed 13 haplotypes, and haplotype diversity of 0.9 (Fig 3A). All of the haplotypes were host-specific, and within two host species, multiple haplotypes were observed. Five haplotypes each were observed in *R*. *satarae* (with two distinct lineages RS1 and RS2) and synanthropic species *M*. *musculus* (with another two distinct lineages MM1 and MM2). Both of these species had a high prevalence among the common species found in the landscape. Among the five haplotypes identified from *M*. *musculus*, four were closely related, and three of them (satellite haplotypes) originated from a central haplotype. The sequence from the pooled mites from *M*. *musculus* also belonged to this central haplotype. The fifth haplotype from *M*. *musculus* (MM2) was distinct and topologically close to RS2 lineage. Sequences from *M*. *booduga*, *F*. *tristriatus*, and *S*. *murinus* formed three independent haplotypes MB1, FT1, and SM1, respectively. The sequence obtained from mites collected from *M*. *booduga* also belonged to the MB1 haplotype. The results of the haplotype network were used to create a haplotype accumulation curve (Fig 3B). The shape of the curve indicates that there is a significant turnover of *Bartonella* haplotypes in the small mammals sampled. The extrapolated region of the haplotype accumulation curve revealed three more possible haplotypes, with the curve reaching the asymptote at around 160 positive individuals. Additional sampling of 100 positive individuals is estimated to capture all the haplotypes present in the small mammals. This indicates that we captured the majority (81%) of the haplotype diversity at the sampling sites.

### Phylogenetic position of small mammal-associated *Bartonella*

**Fig 4:**
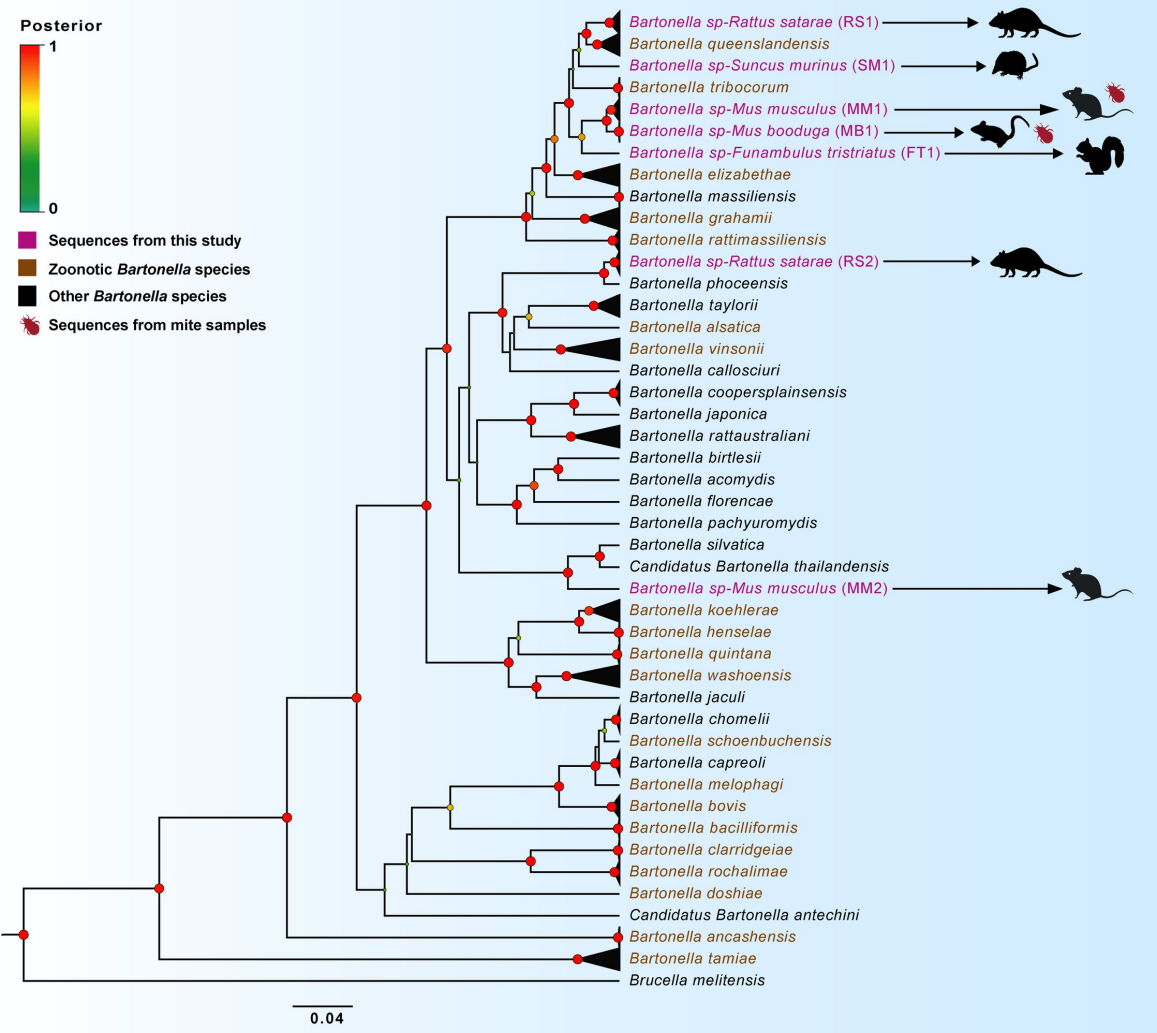
Bayesian phylogenetic inference of *Bartonella* based on 773 bp of *rpoB* sequences. Sequences from this study, zoonotic *Bartonella* and other *Bartonella* are colored purple, brown, and black respectively. Nodes are colored based on posterior probability and monophyletic clades are collapsed for easy visualization. The tree was rooted with one representative of *Brucella melitensis*. The original phylogenetic tree and accession numbers (MT787669-MT787733) are provided in the S1 Fig and S2 Table respectively. The silhouette images of animals were downloaded from PhyloPic (http://phylopic.org), an open-access database that stores reusable silhouette images of organisms.

The phylogenetic analysis based on 773 bp of the *rpoB* locus revealed seven distinct lineages of *Bartonella*. The 65 sequences from this study (GenBank accession no. MT787669-MT787733) were polyphyletic and phylogenetically related to multiple *Bartonella* species. (Fig 4). Sequences from *R*. *satarae* formed two independent monophyletic lineages, with the first one (RS1) being a sister clade to *B*. *queenslandensis* (PP = 1). The second lineage (RS2) formed a sister lineage to *B*. *phoceensis* (PP = 1). Sequences from *M*. *musculus* also formed two lineages, the first one (MM1) as a sister clade (PP = 1) to sequences from *M*. *booduga* and its mites (MB1). Sequence from *F*. *tristriatus* (FT1) was distinct and formed an outgroup to this *M*. *musculus—M*. *booduga* clade (MM1 and MB1). These three lineages formed a monophyletic clade and were nested between *B*. *tribocorum* and *B*. *elizabethae* (PP = 1). The sequence from the pooled mites collected from *M*. *musculus* was also part of the above-mentioned clade (S1 Fig). The second lineage from *M*. *musculus* (MM2) formed an outgroup to Candidatus *B*. *thailandensis—B*. *sylvatica* lineage (PP = 1). Finally, the single sequence from *S*. *murinus* (SM1) was nested between RS1—*B*. *queenslandensis* lineage and *B*. *tribocorum*, but with low posterior probability support (PP <0.7).

The multi-locus phylogeny based on 1861 bp of *rpoB*, *ftsZ*, and *16SrRNA* showed a concordant topology to the *rpoB* phylogeny with few minor deviations (S2 Fig). Divergence of the RS2 lineage into two was one such deviation, although this had low posterior probability support (PP <0.5). Another difference was that the SM1 lineage formed a sister lineage to *B*. *tribocorum* (PP = 0.7) but was nested between *B*. *tribocorum* and *B*. *queenslandensis* in *rpoB* phylogeny. Similarly, the FT1 lineage formed an outgroup to the *B*. *tribocorum—*SM1 lineage (PP = 0.8) whereas the same was an outgroup to the MM1-MB1 lineage in *rpoB* phylogeny. However, these minor deviations did not alter the phylogenetic topology or interpretations. Moreover, the posterior probability support for many lineages in the multi-locus phylogeny was considerably lower, hence the *rpoB* phylogeny was primarily used to draw phylogenetic conclusions in this study.

## Discussion

We investigated the prevalence, diversity, and phylogenetic affinity of *Bartonella* in a small mammal community in the Western Ghats biodiversity hotspot. We report *Bartonella* from five species of small mammals and small mammal-associated mites. This is the first time that *Bartonella* was documented in three of these species—*Rattus satarae*, *Mus booduga*, and *Funambulus tristriatus*. The results from spleen and dried blood spots were nearly identical. We expected a similar prevalence in dried blood spots as *Bartonella* species are known to present in the blood of natural hosts [46]. Also, the larger volume of template DNA (4 μl as opposed to 2 μl used for spleen samples) from these samples may have increased the sensitivity of PCR detection. We speculate this could be the reason we have nearly identical results from spleen and dried blood spots. Every negative control used for each PCR run was negative, indicating there was no issue with contamination. Similar patterns are observed with other tropical pathogens using dried blood spots as a sensitive alternative to other gold standard sample types [47,48].

### High prevalence of *Bartonella* in the small mammal community

The results of the study confirmed our predictions of overall prevalence in the host community and high prevalence in synanthropic species. The high prevalence in the host community (52.48) is consistent with other studies from tropical countries [22,49]. However, the overall prevalence estimate may be affected by the oversampling of common host species [50], such as the large sample size of *R*. *satarae* and *M*. *musculus*. Targeted sampling of rare species could address this bias [51]. For individual species, prevalence patterns varied. Among the four common species found in the landscape, the highest prevalence was observed in *R*. *satarae* followed by *M*. *musculus*. The former is known to prefer forested habitats including plantations and forest edges [52,53]. The latter is a widely distributed synanthrope [54] captured in forests and grasslands also known to show preference to a wide range of habitat types [52]. While the detection of *Bartonella* in *R*. *satarae* was novel, the high prevalence was not surprising since other members of *Rattus* genus are known to host *Bartonella* with high prevalence [49]. In contrast, *R*. *rattus*, another member from this genus and a well-known synanthrope [53] with previous reports of *Bartonella* infection [22], were negative. This result could be due to the low sample size of *R*. *rattus* in this study [55]. The high prevalence of *Bartonella* in *R*. *satarae* and *M*. *musculus* are of concern as they are most common in forested and abandoned plantations, often close to human habitations [56]. As human-altered habitats tend to allow generalist and synanthropic species to reach high abundance, they can enhance the transmission risk of *Bartonella* and other zoonotic pathogens to several incidental hosts, including humans [57,58]. Of the four synanthropic species in this study (*R*. *rattus*, *M*. *musculus*, *M*. *booduga*, and *Suncus murinus*), *Bartonella* was detected in three species (*M*. *musculus*, *M*. *booduga*, and *S*. *murinus*). Two of these species (*M*. *booduga* and *S*. *murinus*) were captured in human habitations. This indicates the presence of *Bartonella* in human habitation. However, low sample sizes for these species from human habitations precludes us from making any comparisons with other land-use types. Future studies with more focused sampling to capture synanthropic species could help discern any patterns in prevalence if present.

### Under detected *Bartonella* diversity in the Western Ghats small mammals

Even though *Bartonella* was detected in only five small mammal species, the haplotype network analysis revealed high diversity. As expected, the bulk of this diversity was contributed by *R*. *satarae* and *M*. *musculus*, the two species detected with high prevalence in the community. Understanding the existing genetic diversity of *Bartonella* in this community is essential as the two host species (*M*. *musculus* and *S*. *murinus*) are widespread synanthropes and are also known to carry zoonotic species of *Bartonella* [4]. Furthermore, it is crucial from a public health perspective, as diversity may impact the evolution of high virulence, novel infections, and drug-resistant strains [59]. *Bartonella* haplotypes identified from mites were not novel and were identical to their respective hosts (*M*. *musculus and M*. *booduga*). Hematophagous ectoparasites are known to acquire these bacteria from their hosts and also transmit them to other incidental hosts [4]. However, the vector competence of these arthropods from this study is unknown. Our interpretation of diversity here remains limited due to the smaller sequence coverage used in this study relative to the *Bartonella* genome. Therefore, generating whole-genome data for these genetic variants will provide comprehensive insights into existing diversity and its implications for public health [60].

Due to lower rank abundance in the host community, our sample sizes for species such as *F*. *tristriatus* and *S*. *murinus* (and mite samples) are low. This made it difficult for us to compare the genetic diversity across species and land-use types. Interestingly, the haplotype accumulation curves suggest that a larger sample size of positive individuals might capture a few more novel *Bartonella* haplotypes from small mammals. This further substantiates our hypothesis that uncaptured *Bartonella* diversity exists in small mammals in the mixed-use landscapes of the Western Ghats. As a biodiversity hotspot with high human density and mammalian richness, pathogen diversity and potential zoonoses are predicted to be high for this landscape [61,62]. Large spatial scale sampling across the Western Ghats is necessary to understand the true *Bartonella* diversity and further validate these predictions. Such surveillance efforts targeting host and vector communities will help lay the foundation for understanding geographical risk, host associations, and natural transmission cycles of these pathogens. Additionally, investigating these pathogens in humans and comparison of data with other hosts and vectors will hold a clue to many febrile illnesses prevailing in forested areas in this biodiversity hotspot.

### Potential zoonotic lineages of *Bartonella* in the small mammal community

Both the haplotype network and the phylogenetic results support the existence of multiple *Bartonella* lineages in small mammals. The most intriguing is the existence of divergent lineages in a single host species. Earlier studies have observed multiple lineages with independent genetic affinities in single host species, particularly in the genus *Rattus* [22,49]. Bai et al. [63] demonstrated this genetic heterogeneity of *Bartonella* sequences from *Rattus* with 23 unique variants falling into six different lineages. In our study, one of the *Bartonella* lineages from *R*. *satarae* (RS1) showed phylogenetic similarity to *B*. *queenslandensis* and the other (RS2) to *B*. *phoceensis*. Frank et al. [3] reported genetic similarity of *Bartonella* sequences reported from a febrile patient in Thailand to *B*. *queenslandensis*, a species previously isolated from Australian and Asian rodents [22]. Sequences from synanthropic species *S*. *murinus* showed phylogenetic relatedness to *B*. *queenslandensis* and *B*. *tribocorum*. Similarly, *Bartonella* lineages derived from *M*. *musculus*, *M*. *booduga*, and *F*. *tristriatus* were phylogenetically related to *B*. *tribocorum* and *B*. *elizabethae*. Both *B*. *tribocorum* and *B*. *elizabethae* were previously detected in small mammals and febrile illness patients in Asia as well as Europe [64,65]. Hence, the phylogenetic relatedness of five *Bartonella* lineages from this study (RS1, MM1, MB1, FT1, and SM1) to these zoonotic species indicates that these lineages could also potentially be zoonotic. Since the *Bartonella* sequences from pooled mites were also part of these zoonotic lineages, these arthropods may be involved in the transmission. Whole-genome analysis and pathogenicity prediction using computational tools might substantiate these phylogenetic signals from these zoonotic lineages [66]. Furthermore, additional sampling of vectors and identification of their species will aid in understanding the transmission interfaces of *Bartonella* across species and land-use types.

In summary, we attempt to understand the prevalence, haplotype diversity, and genetic affinity of *Bartonella* in India. Several lineages of *Bartonella* identified from small mammals including synanthropic species are phylogenetically related to known zoonotic *Bartonella* species. The detection of mites carrying these haplotypes substantiates the possibility of mite-mediated transmission to other incidental hosts including humans, heightening the risk of bartonellosis in the landscape. The presence of diverse *Bartonella* lineages in common rodent species, such as *R*. *satarae* and *M*. *musculus* highlights suspected reservoirs that future research must monitor. Zoonotic lineages identified from widespread synanthropic species need to be further investigated to understand the urban and peri-urban cycles of these pathogens. Despite the small spatial coverage, our results are significant in the context of emerging zoonotic infections and public health. Our results on these under-detected emerging pathogens reiterate the importance of strengthened surveillance in such areas, especially given the importance of global trade, travel, and pathogen movements across continents.

## Supporting information

S1 Table Primers used for multi-locus sequencing in this study. (DOCX)

S2 Table GenBank accession numbers of sequences reported from this study. (DOCX)

S1 Fig Bayesian phylogenetic inference of *Bartonella* based on 773 bp of *rpoB* sequences. Sequences from this study, zoonotic *Bartonella*, and other *Bartonella* are colored purple, brown, and black respectively. The tree was inferred by gene partitioned analysis using BEAST 1.10.0 with 10^8^ MCMC iterations. The posterior probability for the nodes is colored based on the values and monophyletic clades are collapsed for easy visualization. The tree was rooted with one representative of *Brucella melitensis*. The accession numbers for the sequences are provided in the S2 Table. The silhouette images of animals were downloaded from PhyloPic (http://phylopic.org), an open-access database that stores reusable silhouette images of organisms.
(PDF)

S2 Fig Bayesian phylogenetic inference of *Bartonella* based on 1861 bp of multi-locus (*rpoB*, *ftsZ*, and *16SrRNA*) sequences. Sequences from this study, zoonotic *Bartonella* and other *Bartonella* are colored purple, brown, and black respectively. The tree was inferred by gene partitioned analysis using BEAST 1.10.0 with 10^8^ MCMC iterations. The posterior probability for the nodes is colored based on the values, and monophyletic clades are collapsed for easy visualization. The tree was rooted with one representative of *Brucella melitensis*. The accession numbers for the sequences are provided in S2 Table. The silhouette images of animals were downloaded from PhyloPic (http://phylopic.org), an open-access database that stores reusable silhouette images of organisms.
(PDF)

S1 File *Bartonella* screening results. This file lists all individuals used in this study, their screening results, and the land-use type where the individuals were captured.
(XLSX)
