# Supplementary material for: High prevalence and diversity of Bartonella in small mammals from the biodiverse Western Ghats
Source: PLoS Negl Trop Dis. 2021 Mar 11;15(3):e0009178. doi: 10.1371/journal.pntd.0009178 (PMC7951854; doi:10.1371/journal.pntd.0009178)
Supplement: S1 Table — (DOCX) [file pntd.0009178.s001.docx]

**S1 Table. Primers used for multi-locus sequencing in this study**

| Target | Primer sequence (5’-3’) | Size (bp) | Annealing temperature | Reference |
| --- | --- | --- | --- | --- |
| *rpoB* | 1400F: CGCATTGGCTTACTTCGTATG | 825 | 53°C | [1] |
|  | 2300R: GTAGACTGATTAGAACGCTG |  |  |  |
| *ftsZ* | Bfp1: ATTAATCTGCAYCGGCCAGA | ~900 | 56°C* | [2] |
|  | Bfp2: ACVGADACACGAATAACACC |  |  |  |
| *16S rRNA* | 16SF: TCAGAACGAACGCTGGCGGC | 369 | 54°C* | [3] |
|  | 16SR: CGTCATTATCTTCACCGG |  |  |  |

*A touch down protocol was used to avoid non-specific amplification: Starting annealing temperature was 10°C greater than the original annealing temperature and reduction of 1°C at each cycle till the original annealing temperature is reached.

**References**

1. Renesto P, Gouvernet J, Drancourt M, Roux V, Raoult D. Use of rpoB Gene Analysis for Detection and Identification of Bartonella Species. J Clin Microbiol. 2001;39: 430–437. doi:10.1128/JCM.39.2.430-437.2001

2. Zeaiter Z, Liang Z, Raoult D. Genetic Classification and Differentiation of Bartonella Species Based on Comparison of Partial ftsZ Gene Sequences. J Clin Microbiol. 2002;40: 3641–3647. doi:10.1128/JCM.40.10.3641-3647.2002

3. Paziewska A, Harris PD, Zwolińska L, Bajer A, Siński E. Recombination Within and Between Species of the Alpha Proteobacterium Bartonella Infecting Rodents. Microb Ecol. 2011;61: 134–145. doi:10.1007/s00248-010-9735-1
