# Supplementary material for: High prevalence and diversity of Bartonella in small mammals from the biodiverse Western Ghats
Source: PLoS Negl Trop Dis. 2021 Mar 11;15(3):e0009178. doi: 10.1371/journal.pntd.0009178 (PMC7951854; doi:10.1371/journal.pntd.0009178)
Supplement: S2 Table — (DOCX) [file pntd.0009178.s002.docx]

**S2 Table. GenBank accession numbers of sequences reported from this study**

| **Sl. No.** | **Sequence ID** | **Accession No.** | | |
| --- | --- | --- | --- | --- |
|  |  | ***rpoB*** | ***ftsZ*** | ***16Sr RNA*** |
| 1 | Mite-*Mus booduga*-NCBS.KDM17.001 | MT787669 | MT790780 | MT785315 |
| 2 | Mite-*Mus musculus*-NCBS.KDM17.002 | MT787670 | MT790781 | MT785316 |
| 3 | *Funambulus tristriatus*-NCBS.KDM17.040 | MT787671 | MT790782 | MT785317 |
| 4 | *Rattus satarae*-NCBS.KDM18.001 | MT787672 | MT790783 | MT785318 |
| 5 | *Rattus satarae*-NCBS.KDM18.002 | MT787673 | MT790784 | - |
| 6 | *Rattus satarae*-NCBS.KDM18.003 | MT787674 | MT790785 | - |
| 7 | *Rattus satarae*-NCBS.KDM18.006 | MT787675 | MT790786 | - |
| 8 | *Rattus satarae*-NCBS.KDM18.007 | MT787676 | MT790787 | MT785319 |
| 9 | *Rattus satarae*-NCBS.KDM18.008 | MT787677 | MT790788 | - |
| 10 | *Rattus satarae*-NCBS.KDM18.010 | MT787678 | MT790789 | - |
| 11 | *Rattus satarae*-NCBS.KDM18.011 | MT787679 | MT790790 | MT785320 |
| 12 | *Rattus satarae*-NCBS.KDM18.013 | MT787680 | - | MT785321 |
| 13 | *Rattus satarae*-NCBS.KDM18.015 | MT787681 | MT790791 | - |
| 14 | *Rattus satarae*-NCBS.KDM18.016 | MT787682 | - | MT785322 |
| 15 | *Mus musculus*-NCBS.KDM18.017 | MT787683 | MT790792 | MT785323 |
| 16 | *Rattus satarae*-NCBS.KDM18.018 | MT787684 | MT790793 | MT785324 |
| 17 | *Rattus satarae*-NCBS.KDM18.019 | MT787685 | MT790794 | MT785325 |
| 18 | *Rattus satarae*-NCBS.KDM18.021 | MT787686 | MT790795 | - |
| 19 | *Rattus satarae*-NCBS.KDM18.024 | MT787687 | MT790796 | MT785326 |
| 20 | *Rattus satarae*-NCBS.KDM18.026 | MT787688 | MT790797 | - |
| 21 | *Rattus satarae*-NCBS.KDM18.027 | MT787689 | MT790798 | MT785327 |
| 22 | *Rattus satarae*-NCBS.KDM18.028 | MT787690 | MT790799 | - |
| 23 | *Rattus satarae*-NCBS.KDM18.029 | MT787691 | - | MT785328 |
| 24 | *Rattus satarae*-NCBS.KDM18.030 | MT787692 | - | MT785329 |
| 25 | *Rattus satarae*-NCBS.KDM18.032 | MT787693 | MT790800 | - |
| 26 | *Mus booduga*-NCBS.KDM18.034 | MT787694 | MT790801 | MT785330 |
| 27 | *Rattus satarae*-NCBS.KDM18.035 | MT787695 | MT790802 | MT785331 |
| 28 | *Rattus satarae*-NCBS.KDM18.036 | MT787696 | - | MT785332 |
| 29 | *Rattus satarae*-NCBS.KDM18.037 | MT787697 | - | MT785333 |
| 30 | *Rattus satarae*-NCBS.KDM18.038 | MT787698 | MT790803 | MT785334 |
| 31 | *Rattus satarae*-NCBS.KDM18.039 | MT787699 | - | MT785335 |
| 32 | *Rattus satarae*-NCBS.KDM18.042 | MT787700 | - | - |
| 33 | *Rattus satarae*-NCBS.KDM18.044 | MT787701 | MT790804 | - |
| 34 | *Rattus satarae*-NCBS.KDM18.045 | MT787702 | MT790805 | MT785336 |
| 35 | *Rattus satarae*-NCBS.KDM18.047 | MT787703 | - | MT785337 |
| 36 | *Rattus satarae*-NCBS.KDM18.048 | MT787704 | MT790806 | MT785338 |
| 37 | *Rattus satarae*-NCBS.KDM18.049 | MT787705 | MT790807 | MT785339 |
| 38 | *Rattus satarae*-NCBS.KDM18.050 | MT787706 | MT790808 | MT785340 |
| 39 | *Rattus satarae*-NCBS.KDM18.054 | MT787707 | MT790809 | MT785341 |
| 40 | *Rattus satarae*-NCBS.KDM18.055 | MT787708 | - | MT785342 |
| 41 | *Rattus satarae*-NCBS.KDM18.056 | MT787709 | MT790810 | MT785343 |
| 42 | *Rattus satarae*-NCBS.KDM18.057 | MT787710 | MT790811 | MT785344 |
| 43 | *Rattus satarae*-NCBS.KDM18.058 | MT787711 | MT790812 | MT785345 |
| 44 | *Rattus satarae*-NCBS.KDM18.059 | MT787712 | MT790813 | - |
| 45 | *Rattus satarae*-NCBS.KDM18.061 | MT787713 | MT790814 | - |
| 46 | *Rattus satarae*-NCBS.KDM18.062 | MT787714 | - | - |
| 47 | *Rattus satarae*-NCBS.KDM18.064 | MT787715 | MT790815 | MT785346 |
| 48 | *Rattus satarae*-NCBS.KDM18.066 | MT787716 | MT790816 | MT785347 |
| 49 | *Rattus satarae*-NCBS.KDM18.067 | MT787717 | MT790817 | MT785348 |
| 50 | *Rattus satarae*-NCBS.KDM18.068 | MT787718 | MT790818 | MT785349 |
| 51 | *Mus musculus*-NCBS.KDM18.070 | MT787719 | MT790819 | - |
| 52 | *Mus musculus*-NCBS.KDM18.071 | MT787720 | MT790820 | MT785350 |
| 53 | *Mus musculus*-NCBS.KDM18.081 | MT787721 | MT790821 | MT785351 |
| 54 | *Mus musculus*-NCBS.KDM18.082 | MT787722 | MT790822 | MT785352 |
| 55 | *Mus musculus*-NCBS.KDM18.089 | MT787723 | MT790823 | - |
| 56 | *Mus musculus*-NCBS.KDM18.092 | MT787724 | MT790824 | MT785353 |
| 57 | *Mus musculus*-NCBS.KDM18.099 | MT787725 | MT790825 | MT785354 |
| 58 | *Mus musculus*-NCBS.KDM18.102 | MT787726 | MT790826 | MT785355 |
| 59 | *Mus musculus*-NCBS.KDM18.104 | MT787727 | MT790827 | MT785356 |
| 60 | *Mus musculus*-NCBS.KDM18.105 | MT787728 | MT790828 | MT785357 |
| 61 | *Mus musculus*-NCBS.KDM18.106 | MT787729 | MT790829 | MT785358 |
| 62 | *Mus musculus*-NCBS.KDM18.122 | MT787730 | MT790830 | MT785359 |
| 63 | *Rattus satarae*-NCBS.KDM18.126 | MT787731 | MT790831 | - |
| 64 | *Suncus muninus*-NCBS.KDM18.133 | MT787732 | MT790832 | MT785360 |
| 65 | *Rattus satarae*-NCBS.KDM18.134 | MT787733 | MT790833 | MT785361 |
