# Supplementary material for: High prevalence and diversity of Bartonella in small mammals from the biodiverse Western Ghats
Source: PLoS Negl Trop Dis. 2021 Mar 11;15(3):e0009178. doi: 10.1371/journal.pntd.0009178 (PMC7951854; doi:10.1371/journal.pntd.0009178)
Supplement: S2 Fig — Sequences from this study, zoonotic Bartonella and other Bartonella are colored purple, brown, and black respectively. The tree was inferred by gene partitioned analysis using BEAST 1.10.0 with 108 MCMC iterations. The posterior probability for the nodes is colored based on the values, and monophyletic clades are collapsed for easy visualization. The tree was rooted with one representative of Brucella melitensis. The accession numbers for the sequences are provided in S2 Table. The silhouette images of animals were downloaded from PhyloPic (http://phylopic.org), an open-access database that stores reusable silhouette images of organisms. (PDF) [file pntd.0009178.s004.pdf]

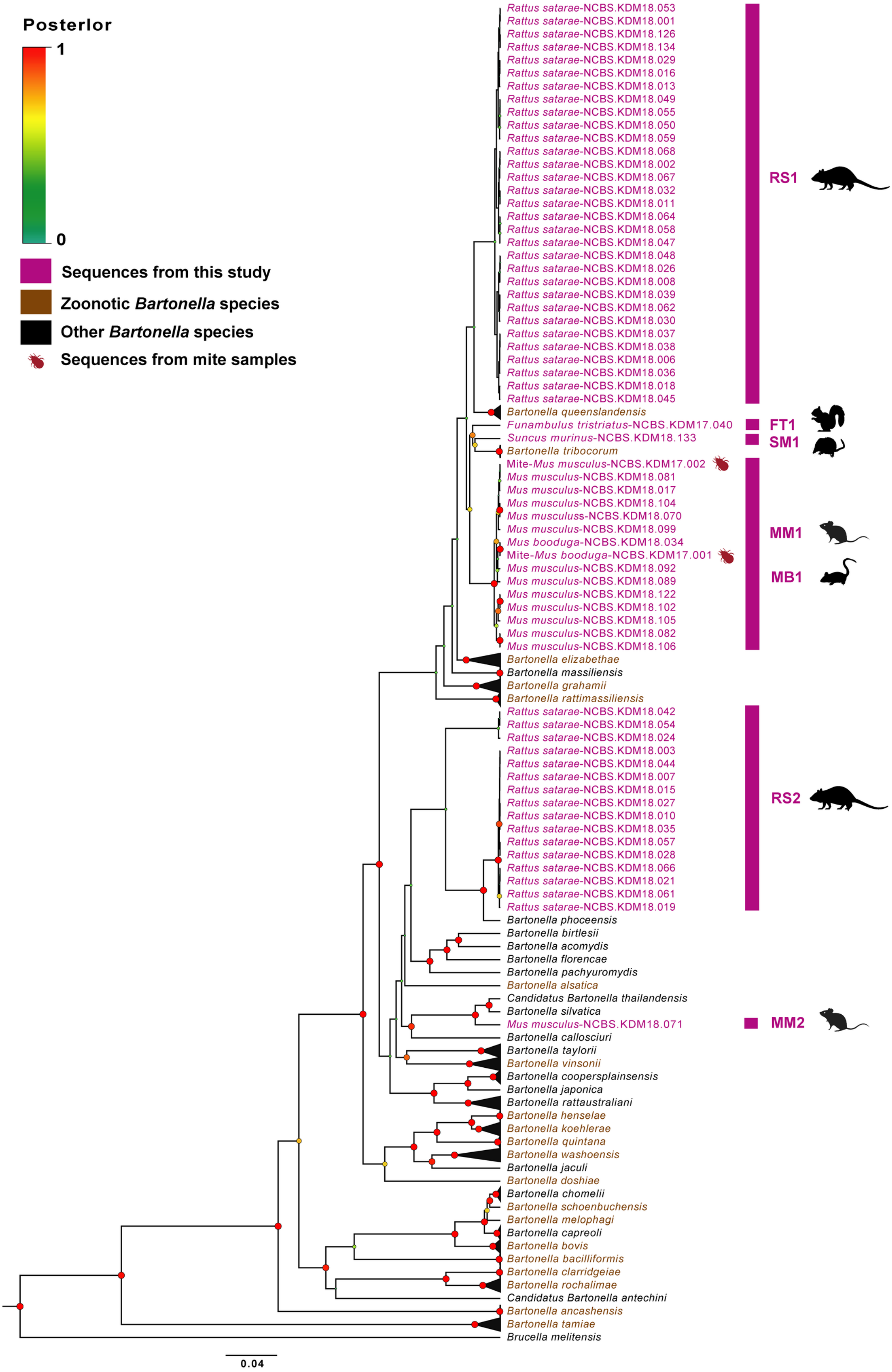
Posterlor
1
0
Sequences from this study
Zoonotic *Bartonella* species
Other *Bartonella* species
Sequences from mite samples
Rattus satarae-NCBS.KDM18.053
Rattus satarae-NCBS.KDM18.001
Rattus satarae-NCBS.KDM18.126
Rattus satarae-NCBS.KDM18.134
Rattus satarae-NCBS.KDM18.029
Rattus satarae-NCBS.KDM18.016
Rattus satarae-NCBS.KDM18.013
Rattus satarae-NCBS.KDM18.049
Rattus satarae-NCBS.KDM18.055
Rattus satarae-NCBS.KDM18.050
Rattus satarae-NCBS.KDM18.059
Rattus satarae-NCBS.KDM18.068
Rattus satarae-NCBS.KDM18.002
Rattus satarae-NCBS.KDM18.067
Rattus satarae-NCBS.KDM18.032
Rattus satarae-NCBS.KDM18.011
Rattus satarae-NCBS.KDM18.064
Rattus satarae-NCBS.KDM18.058
Rattus satarae-NCBS.KDM18.047
Rattus satarae-NCBS.KDM18.048
Rattus satarae-NCBS.KDM18.026
Rattus satarae-NCBS.KDM18.008
Rattus satarae-NCBS.KDM18.039
Rattus satarae-NCBS.KDM18.062
Rattus satarae-NCBS.KDM18.030
Rattus satarae-NCBS.KDM18.037
Rattus satarae-NCBS.KDM18.038
Rattus satarae-NCBS.KDM18.006
Rattus satarae-NCBS.KDM18.036
Rattus satarae-NCBS.KDM18.018
Rattus satarae-NCBS.KDM18.045
Bartonella queenslandensis
Funambulus tristriatus-NCBS.KDM17.040
Suncus murinus-NCBS.KDM18.133
Bartonella tribocorum
Mite-*Mus musculus*-NCBS.KDM17.002
Mus musculus-NCBS.KDM18.081
Mus musculus-NCBS.KDM18.017
Mus musculus-NCBS.KDM18.104
Mus musculuss-NCBS.KDM18.070
Mus musculus-NCBS.KDM18.099
Mus booduga-NCBS.KDM18.034
Mite-*Mus booduga*-NCBS.KDM17.001
Mus musculus-NCBS.KDM18.092
Mus musculus-NCBS.KDM18.089
Mus musculus-NCBS.KDM18.122
Mus musculus-NCBS.KDM18.102
Mus musculus-NCBS.KDM18.105
Mus musculus-NCBS.KDM18.082
Mus musculus-NCBS.KDM18.106
Bartonella elizabethae
Bartonella massiliensis
Bartonella grahamii
Bartonella rattimassiliensis
Rattus satarae-NCBS.KDM18.042
Rattus satarae-NCBS.KDM18.054
Rattus satarae-NCBS.KDM18.024
Rattus satarae-NCBS.KDM18.003
Rattus satarae-NCBS.KDM18.044
Rattus satarae-NCBS.KDM18.007
Rattus satarae-NCBS.KDM18.015
Rattus satarae-NCBS.KDM18.027
Rattus satarae-NCBS.KDM18.010
Rattus satarae-NCBS.KDM18.035
Rattus satarae-NCBS.KDM18.057
Rattus satarae-NCBS.KDM18.028
Rattus satarae-NCBS.KDM18.066
Rattus satarae-NCBS.KDM18.021
Rattus satarae-NCBS.KDM18.061
Rattus satarae-NCBS.KDM18.019
Bartonella phoceensis
Bartonella birtlesii
Bartonella acomydis
Bartonella florencae
Bartonella pachyuromydis
Bartonella alsatica
Candidatus *Bartonella thailandensis*
Bartonella silvatica
Mus musculus-NCBS.KDM18.071
Bartonella callosciuri
Bartonella taylorii
Bartonella vinsonii
Bartonella coopersplainsensis
Bartonella japonica
Bartonella rattaustraliani
Bartonella henselae
Bartonella koehlerae
Bartonella quintana
Bartonella washoensis
Bartonella jaculi
Bartonella doshiae
Bartonella chomelii
Bartonella schoenbuchensis
Bartonella melophagi
Bartonella capreoli
Bartonella bovis
Bartonella bacilliformis
Bartonella clarridgeiae
Bartonella rochalimae
Candidatus *Bartonella antechini*
Bartonella ancashensis
Bartonella tamiae
Brucella melitensis
RS1
FT1
SM1
MM1
MB1
RS2
MM2
0.04
